# Technic of Administering Nitrous Oxide and Oxygen to the Average Adult Patient*Extracted from a paper read before the National Dental Association, and printed in the July *Journal of the National Dental Association*, in full, with illustrations.

**Published:** 1919-08

**Authors:** J. P. Henahan


### TECHNIC OF ADMINISTERING NITROUS OXIDE AND OXYGEN TO THE AVERAGE ADULT PATIENT *

BY J. P. HENAHAN, D.D.S.

At the time when a patient is to be given an anesthetic in the dentist's chair, it is supposed that as far as possible all preliminary arrangements have been made.

---

* Extracted from a paper read before the National Dental Association, and printed in the July *Journal of the National Dental Association,* in full, with illustrations.

The patient has been fasting for three hours at least before the appointed hour.

If the patient is a woman she should be left for a short time to the attentions of the lady assistant who gives advice which precludes any embarrassing situation during anesthesia.

All tight collars, neck bands, or excessively tight body lacing should be removed.

It is to be supposed, whether evident or not, that the patient is in a state of high nervous tension.

The patient should be placed in a comfortable position in the chair.

The dentist by action and conversation applies suggestion calculated to quiet the fears of the patient, because as stated before, successful technic for the administration of nitrous oxide and oxygen must comprise also to a great extent treatment of the mind.

The patient should be shown the easiest position in which to sit.

The feet should be extended, but not shoving, against the foot-rest.

The hands should be resting in the lap, not grasping the arms of the chair.

The neck should not be bent backward, because this excites a desire to swallow constantly.

It should not be bent forward because, especially after anesthesia develops, it interferes with free respiration, because the chin approaches or rests upon the chest, constricting the air passages in the throat.

The aim is to have the patient in a comfortable position and then have patient relax completely; pay some attention to this, for if the patient remains tense with muscles contracted, the tendency is to become more so, requiring a much deeper and more prolonged anesthetic.

At the most favorable moment the patient is again reassured, in a very positive manner, that there is no danger and that no pain will be experienced.

Notice privately that your patient breathes comfortably through the nose with the lips closed, for many patients believe, without cause, that they can not breathe through the nose.

You have determined beforehand what work is to be performed, and then you carefully select a mouth prop which will hold the mouth open sufficiently to permit operation, but not to such an uncomfortable extent that it causes swallowing or interference with respiration. Some types present in whom the lower jaw is short due either to lack of development, or as the result of scalds or burns. In the latter case the cicatricial tissue binds the jaw and limits its action. In any of these cases there is a tendency toward automatic closure of the air passages when an attempt is made to open the mouth to any great extent. Extreme care must be exercised and intermissions permitted to allow for correction of any respiratory interference after anesthesia is induced.

It will be noticed in many operations upon the lower jaw, that a tendency toward cyanosis will develop; this is not due, as a rule, to incorrect mixture of gas and oxygen, but to partial closure of air passage due to pressure necessary in operation. This condition is eliminated by frequent intermissions and drawing forward of the jaw.

When all preliminary details have been arranged, place a mouth prop of proper size in position and place mouth cover over the mouth and permit the patient to continue breathing as usual; inform him then that he is doing just what you want him to do, that he is breathing through the nose.

If the patient shows fear or undue excitement at this stage and the manner of breathing is affected, being either too shallow or violent, say quietly that comfortably deep and regular breathing is what you desire; inform your patient not to talk or swallow. The object is to avoid a feeling of suffocation.

Now, examine your nasal inhaler and note that the expiratory and air valves are wide open. This is a very important detail; place your inhaler quietly and carefully on the patient's nose, and after allowing a few moments for patient to become accustomed to it, carefully and slowly turn in nitrous oxide gas.

It may be remembered that the patient will, on account of the open condition of the inhaler, be breathing with no more difficulty than before the inhaler was placed, and our object should be not to change conditions suddenly and perhaps cause panic or excitement by allowing gas to rush in suddenly.

As the gas flows into and partly fills the bag, the air valves (not respiratory valves) should be gradually closed. When this time arrives nitrous oxide has been substituted for atmosphere and we must carefully observe our patient for signs which will indicate the necessity for oxygen. It is not possible to give definite rules for this, as conditions vary with all patients; however, the majority will be affected in more or less a common manner.

The average patient after inhaling about six full breaths of nitrous oxide, will show faint signs of approaching cyanosis and should be given oxygen. About 8 per cent. on an average should be admitted at this time; as the anesthesia progresses more oxygen usually must be added. The oxygen must be added according to the developing symptoms of the patient and not according to any set of rules, each patient being a problem in himself.

The anesthetist's ear should be trained to respiratory sounds and it should be remembered that while the patient retains any control of respiration, that anesthesia is not profound.

Under the foregoing conditions, about one minute is required to induce anesthesia, and the patient should present an appearance as if in a state of natural sleep.

The color of the skin should be practically the same as before anesthesia was induced.

Respiration should be profound and measured.

The pupil should not be dilated (should not be more than two or three mm. in circumference).

The pulse should be full, but not noticeably increased either in size or frequency.

### MIXTURE OF GASES

If the mixture of gasses has not been correctly made, the patient will present relative symptoms:

First, for want of sufficient oxygen the patient presents symptoms of a straight nitrous oxide anesthesia, and will become cyanotic, due to partial asphyxia; or,

Secondly, on account of too great a supply of oxygen, will be unduly stimulated.

In the latter case, the patient's color is very red, especially the lips and the lobes of the ears.

The breathing is irregular with possible holding and muscular straining, and if not quickly corrected, the patient becomes excited and possibly troublesome.

If the mixture is correctly made, as evidenced by the fact that the color does not change in one way or the other, but still the patient is not sufficiently anesthetized, then we must modify the technic so as to cause the blood to take up more nitrous oxide. This is done by increasing the inter-pulmonary pressure, in the following way:

The respiratory valve on the inhaler, which up to this time has been working rather freely, is now partly closed, thus constricting the passage for escape of the expired gas, and automatically increasing the internal pressure in the gas system and the alveoli of the lungs, causing an increase in the amount of gas passing through membranes. The general mixture of nitrous oxide and oxygen is not changed only as the progress of anesthesia indicates the necessity of an increase in the supply of oxygen.

### TECHNIC OF ADMINISTRATION OF NITROUS OXIDE AND OXYGEN TO CHILDREN

In the case of a young child, a great deal of judgment is necessary.

Dentists often are called upon by surgeons for aid in minor surgery cases, such as adenectomy and circumcision. For children either of these operations are more serious than extraction cases, and may, through nervous reflexes, cause the development of respiratory troubles, and the younger the child the more likely are these symptoms to be seen. Deep surgical anesthesia must be maintained without interruption.

Children less than five years of age generally are not regarded as proper subjects for this anesthetic.

Children from five to fourteen years seem to present a different problem at all stages between these years.

The very young are difficult to control before anesthesia, and those about twelve to fourteen very difficult after anesthesia is partly induced.

The youngster oftentimes will not submit quietly and therefore must be attended to in a special manner.

After all efforts to induce the child to submit quietly have failed, the nitrous oxide and oxygen is allowed to commence flowing into the machine. The air valve on inhaler in this case is not opened.

The oxygen is admitted in bountiful proportions from the beginning, and then the inhaler is placed over nose and mouth at once, and no matter whether the child inhales through the nose or mouth or cries out, the gas is admitted to the lungs.

Anesthesia develops quickly and then the child is placed in a correct anesthetic position, and if the oxygen supply is noticeably too great it is gradually reduced.

If the child voluntarily submits, then the technic is practically the same as for the adult. Young children, however, as a rule, breathe more freely through their mouth after it is open, therefore it is advisable to place the inhaler over the mouth until anesthesia is induced, at which time it may be placed over the nose. It is well also to admit oxygen to the mixture plentifully and early, because often asphyxia develops so quickly and suddenly that symptoms are seen even before anesthesia, and are attended by violent muscular contractions of a general character.

Discernment is necessary at this stage for the reason that inexperience may misinterpret these muscular movements as voluntary; it would be a serious mistake to continue without oxygen.

A liberal supply of oxygen prolongs the induction period and prevents the development of the foregoing symptoms.

### MOUTH BREATHING

Much difficulty is encountered in certain cases, either young or old, when the operative stage is reached, because the patient habitually breathes through the mouth and returns to consciousness. This is overcome in either of three ways after the mouth cover is removed:

First, by completely closing the expiratory valve on inhaler, and, if necessary, increasing the volume and pressure of gas, thereby forcing down the soft palate; or,

Second, by manipulating the tongue with the fingers, thereby increasing the tendency to breathe through the nose.

Third, by placing in the dorsum of the mouth a long folded and stitched gauze sponge which prevents the passage of air through the mouth. In the later cases the respiratory valve is left open.

### VOMITING AND NAUSEA

Vomiting, although not occurring often, may be one of the most annoying conditions which we encounter. If vomiting actually occurs during any stage of anesthesia, and the mouth and throat is filled with solid food particles, it is wise to discontinue the anesthetic for a few moments, allowing time to remove all food debris from the air passages.

Vomiting during anesthesia is possible only when anesthesia is light. When the tendency toward vomiting is noticed, anesthesia should be deepened, because light anesthesia stimulates the vomiting center.

Nausea after anesthesia is always the result either of too recently partaking of food, or as will more frequently be the case, when patient is afflicted with some form of stomach trouble. In practically all cases of post-operative nausea, when nitrous oxide and oxygen has been used, a history of easily induced nausea will be discovered.

### DIFFICULT CASES

A certain percentage of what may in passing be termed difficult cases will present in our practice. They usually require application of special technic to produce satisfactory results. A great many secondary reasons are found for these conditions. The environment of the patient, occupation and habits exert a marked influence.

It will be commonly noticed that doctors, dentists and trained nurses as a class take anesthetics in which the nervous tension under which they live will be reflected. Locomotive engineers, chauffeurs, firemen, etc., usually after anesthetics narrate some dream of a violent nature in connection with their occupation. Men addicted to the use of great quantities of tobacco and alcoholic beverages, and patients who are addicted to the use of drugs, present the most difficult and troublesome cases which we see. The immediate reason is that the amount of $N_2O$ taken up at ordinary atmospheric pressure is not sufficient to cause anesthesia.

Sometimes we find it advisable to employ some other than a general anesthetic in these cases, but on the other hand occasionally some condition dictates the use of a general anesthetic. The patient who has recently been the victim of some violent accident lives over in his dreams the harrowing incidents, and adds to the difficulties of the anesthetist. In practically all of these cases it will be found that the patient will tolerate but a very small percentage of oxygen, especially in the early stages of anesthesia.

Relaxation does not occur until the anesthetic has been maintained for some time.

A slight degree of cyanosis is necessary if anesthesia is to be maintained; it serves as a guide—however, the degree must not be such as to modify respiration.

### RESTRAINING THE VIOLENT PATIENT

In the administration of general anesthetics, the manner of restraint for the patient who becomes violent is a subject which should receive careful consideration.

When administering a general anesthetic to a patient, we assume responsibility for his well-being.

If the patient is beset by hallucinations which make him violent, we must apply restraint with the object not only of preventing him from doing violence to those present, but also to prevent him from injuring himself.

Occasionally a patient is seen who seems possessed of superhuman strength and it requires the greatest physical effort to prevent the patient from getting out of the chair.

Various methods of restraint have been suggested and tested, including straps for the wrists and ankles and also straps for around the abdomen. I advise against the use of all these, not because they will not restrain the patient, but because it is possible for the patient to injure himself. A narrow strap around the abdomen might easily cause the fracturing of a rib of a patient if he exerts sufficient strain against it. And fracture of the bones of the arms or legs might also result when narrow straps are applied. Any method of restraining by arm holds about the neck are ill-advised; they are never successful and are exceedingly dangerous. It must be remembered that the patient may be partly anesthetized and may easily exert sufficient force to cause injury.

After much experimenting, I have adopted a large linen band of roller-towel material, eight feet in length. This band can be placed over the entire abdomen and breast. It is not necessary that the patient know it is for the purpose of restraint; after the anesthetic has progressed, the towel is knotted behind the chair. I have never known this to fail and I have never had patients complain afterwards of soreness because of its use.

### THE GAS MACHINE

The gas machine should be carefully selected. It should be constructed in such manner that instant control is maintained at all times over both gases.

It is a wrong principle that changes the volume of gases when a change is to be made in the supply of oxygen.

Single-valve machines which do not permit of independent control of the oxygen and nitrous oxide, can not be successfully used in dental practice.

The machine should always be perfect in all its parts. There should be no leaky bags or tubes.

The inhaler should not be worn out nor softened.

Silk-covered tubes should not be permitted. Very often the rubber is completely destroyed, the silk conceals this fact, and it is impossible to administer a good anesthetic under these conditions.

### THE ASSISTANT

An assistant capable of observing and thinking quickly is necessary in the administration of anesthetics. It is not necessary that the assistant shall be an anesthetist; she should not be required at any time to assume responsibility. Her duties should be entirely to carry out quickly and precisely instructions regarding the gas mixtures given by the dentist after he has begun operating.

She should be familiar with the working of the particular gas machine and with the inhaler.

She should understand the importance of preventing leakage of atmosphere into the mixture, or loss of the gas around the inhaler, or through leaky tubes.

She should be instructed to the effect that no matter what the operator does she shall keep the margins of the inhaler in close adaptation to the face.

*Confidence is borne of experience, and as one grows in experience he really finds that, especially for cases of short duration, there will be few contraindications.*

